# Efficient 3D imaging and pathological analysis of the human lymphoma tumor microenvironment using light-sheet immunofluorescence microscopy

**DOI:** 10.7150/thno.86221

**Published:** 2024-01-01

**Authors:** Liting Chen, Jiao Meng, Yao Zhou, Fang Zhao, Yifan Ma, Wenyang Feng, Xingyu Chen, Jin jin, Shimeng Gao, Jianchao Liu, Man Zhang, Aichun Liu, Zhenya Hong, Jiang Tang, Dong Kuang, Liang Huang, Yicheng Zhang, Peng Fei

**Affiliations:** 1 Department of Hematology, Tongji Hospital, Tongji Medical College, Huazhong University of Science and Technology, Wuhan, China.; 2 Hematology Department, Harbin Medical University Cancer Hospital, Harbin Medical University, Harbin, China.; 3 School of Optical and Electronic Information - Wuhan National Laboratory for Optoelectronics, Huazhong University of Science and Technology, Wuhan, China.; 4 Department of Pathology, Tongji Hospital, Tongji Medical College, Huazhong University of Science and Technology, Wuhan, China.

**Keywords:** lymphoma tumor microenvironment, tissue clearing, light-sheet microscopy, three-dimensional imaging, 3D spatial analysis.

## Abstract

**Rationale:** The composition and spatial structure of the lymphoma tumor microenvironment (TME) provide key pathological insights for tumor survival and growth, invasion and metastasis, and resistance to immunotherapy. However, the 3D lymphoma TME has not been well studied owing to the limitations of current imaging techniques. In this work, we take full advantage of a series of new techniques to enable the first 3D TME study in intact lymphoma tissue.

**Methods:** Diverse cell subtypes in lymphoma tissues were tagged using a multiplex immunofluorescence labeling technique. To optically clarify the entire tissue, immunolabeling-enabled three-dimensional imaging of solvent-cleared organs (iDISCO+), clear, unobstructed brain imaging cocktails and computational analysis (CUBIC) and stabilization to harsh conditions via intramolecular epoxide linkages to prevent degradation (SHIELD) were comprehensively compared with the ultimate dimensional imaging of solvent-cleared organs (uDISCO) approach selected for clearing lymphoma tissues. A Bessel-beam light-sheet fluorescence microscope (B-LSFM) was developed to three-dimensionally image the clarified tissues at high speed and high resolution. A customized MATLAB program was used to quantify the number and colocalization of the cell subtypes based on the acquired multichannel 3D images. By combining these cutting-edge methods, we successfully carried out high-efficiency 3D visualization and high-content cellular analyses of the lymphoma TME.

**Results:** Several antibodies, including CD3, CD8, CD20, CD68, CD163, CD14, CD15, FOXP3 and Ki67, were screened for labeling the TME in lymphoma tumors. The 3D imaging results of the TME from three types of lymphoma, reactive lymphocytic hyperplasia (RLN), diffuse large B-cell lymphoma (DLBCL), and angioimmunoblastic T-cell lymphoma (AITL), were quantitatively analyzed, and their cell number, localization, and spatial correlation were comprehensively revealed.

**Conclusion:** We present an advanced imaging-based method for efficient 3D visualization and high-content cellular analysis of the lymphoma TME, rendering it a valuable tool for tumor pathological diagnosis and other clinical research.

## Introduction

Lymphoma refers to a heterogeneous group of tumors and is among the most common types of cancer in the world 1. Lymphomas arise from the clonal proliferation of lymphocytes at different stages of maturation. The latest classification system categorizes lymphomas into B-cell lymphoma (BCL), TCL and NK-cell lymphoma, which originate from different cell types 2. Of all of the lymphoma types, BCL is the most common, accounting for 60-90% of cases, with DLBCL being the most common subtype, accounting for approximately 30% of all non-Hodgkin's lymphomas 3. T-cell lymphoma (TCL) accounts for approximately 12-35% of cases, and the most common subtypes are peripheral TCL (PTCL) and angioimmunoblastic TCL (AITL) 4. Although immunotherapy for lymphoma has recently made great progress, the therapeutic efficiency remains very limited and can be greatly improved 5. An increasing number of studies have indicated that the tumor microenvironment (TME) in lymphomas plays important roles in immunotherapy 6-8.

The TME of lymphoma is heterogeneous and consists largely of tumor cells, immune cells and extracellular matrix 9. The composition of the TME varies widely among lymphoma subtypes. To date, several studies have investigated the TME in BCL and classified the TME of BCL into three main patterns: 'recruitment', exemplified by classical Hodgkin's lymphoma (CHL); 're-education', represented by follicular lymphoma (FL); and 'effacement', observed in Burkitt's lymphoma (BL) 10. The nontumor cells in CHL, FL and BL also vary greatly, accounting for approximately 99%, 50% and 10% of the tumor tissue, respectively. The composition of the TME reflects, to some extent, the proliferation ability of tumor cells and the efficiency of immune cells, which are factors that can help predict the prognosis of lymphoma patients 11. Studies have shown that malignant cells interact with the TME to circumvent immune responses, thereby inducing tumor growth, metastasis, and resistance to immunotherapy 12, 13.

Current techniques for studying the TME include flow cytometry 14, single-cell RNA sequencing (scRNA-seq) 15, spatial transcriptome 16, multiplex immunohistochemistry (mIHC) 17, and image mass cytometry (IMC) 18. Compared to the former two methods, spatial transcriptome, mIHC, and IMC can provide sample spatial information but only for 4-5 µm thin tissue sections. Due to the heterogeneity of tumors, thick tissues containing axial information require the microenvironment to be depicted in three dimensions. Tissue clearing techniques use chemicals to remove the light scattering/attenuation components of the cells while largely retaining their cytoarchitectures, thereby generating an “optically transparent” tissue in its nearly intact status 19, 20. Three major types of clearing strategies, classified as hydrophobic, hydrophilic, and hydrogel-based approaches, have been widely used for processing diverse organ tissues, such as the brain, kidney, heart, lung, pancreas, and even bone 21-27. Grüneboom* et al.* Mapped trans-cortical-vessels (TCVs), arteries and veins using confocal/two-photon laser scanning microscopy (TPLSM) of cleared bones, showed that arterial TCVs could directly feed into the venous circulation at the endosteal surface while a significant part of veins directly fed into bone-crossing TCVs 28. Through the combination of these advanced whole-tissue clearing techniques, light-sheet fluorescence microscopy (LSFM) has emerged, which allows consecutive plane illumination with high speed and a low photobleaching rate 29-31. *intoto* 3D imaging of entire clarified tissues and panoramic investigation of their cytoarchitectures across the whole-tissue level are achieved with this method by using various advanced biomedical applications in histology, pathology and anatomy 19, 32-34. The throughput of traditional optical microscope systems is finite. Whether adopting low-magnification detection to cover a large field of view or high-magnification detection to produce high-resolution images, it is impossible to overcome the limitation of the system's own spatial bandwidth product. However, light-sheet microscopy obtains optical high-throughput, as it adopts wide-field-sheet excitation detection instead of point/line excitation detection, thereby increasing its imaging throughput 35.

In this work, we developed a method containing uDISCO tissue clearing, multiplex immunofluorescence labeling, Bessel-type light-sheet microscopy, and a spatial analysis program to investigate the lymphoma TME, and this method is capable of acquiring a much richer amount of information within a unit of time than other optical systems. We provide comprehensive 3D visualization of the TME across macroscale lymphoma tissues at single-cell resolution. Through the development of a lymphoma TME 3D visualization program, we quantitatively revealed the differences in cell ratios and spatial correlations of numerous immune cell subtypes in the lymphoma TME.

## Materials and methods

### Collection and fixation of fresh human lymph node tissue samples

Three human lymph node specimens obtained postoperatively from Tongji Hospital, Huazhong University of Science & Technology, Wuhan, China, were stored in 4% paraformaldehyde (PFA) fixative solution and fixed overnight at 4 °C. Information on the three patients is listed in Table S1. The fixed lymph node samples were cut into 0.5-1-mm-thick tissue blocks using a vibratome (Leica VT1200, Germany). The dissected tissue blocks were then fixed in 4% PFA at 4 °C until use.

### Validation of antibody compatibility with methanol

Fixed tissue samples were embedded in optimal cutting temperature (OCT) compound and immediately stored at -80 °C. The OCT-embedded tissue was cut into 20 µm frozen sections for antibody validation. Frozen sections were first incubated in 100% methanol for 3 h, rehydrated in PBS and immunostained. Slides not treated with methanol were used as a positive control. Antibodies compatible with methanol treatment were expected to perform well in uDISCO and produce good signal-to-noise ratios (SNRs).

### Sample Pretreatment

All steps were performed in 2 mL Eppendorf tubes. Human lymph node specimens were fixed in 4% PFA at 4 °C, shaken overnight and then moved to room temperature (RT) for 1 h. Fixed tissue blocks were washed three times with 0.1 M PBS for 30 min and then placed into 20%, 40%, 60% and 80% methanol (in ddH2O) in sequence for 1 h at each step and into 100% methanol (MeOH) twice for 1 h for dehydration. Then, the tissue blocks were placed into 66% dichloromethane (DCM)/33% MeOH at RT and shaken overnight. After washing twice in 100% MeOH at RT to remove DCM, tissue blocks were bleached with 5% H2O2 in MeOH (1 Volume 30% H2O2 to 5 volumes MeOH) at 4 °C overnight. After bleaching, the tissue blocks were rehydrated with a MeOH/H2O series, followed by 80%, 60%, 40%, and 20% MeOH for 1 h at each step, PBS for 1 h twice, and finally PBS/0.2% Triton X-100 (PBST) for 1 h twice.

### Immunolabeling

Pretreated tissue blocks were immersed in permeabilization solution (0.2% Triton X-100/20% DMSO/0.3 M glycine in 1X PBS), placed into a constant temperature shaker at 37 °C and gently shaken for 1 d. Next, the tissue was incubated in blocking solution (0.2% Triton X-100/10% DMSO/6% goat serum in 1X PBS) at 37 °C and gently shaken for 1 d. After blocking, the tissue blocks were incubated for 3 d at 37 °C with gentle shaking on a constant temperature shaker in primary antibody dilutions (5% DMSO/3% goat serum in PTwH (0.2% Tween-20 and 10 g/mL heparin in 1X PBS)) and then washed for 10 min, 15 min, 30 min, 60 min, and 120 min in PTwH until the next day. Next, tissue blocks were incubated in secondary antibody dilution (3% goat serum in PTwH) for 3 d at 37 °C with gentle shaking and then washed for 10 min, 15 min, 30 min, 60 min, and 120 min in PTwH until the next day. If nuclear staining was performed, tissue blocks were immersed in propidium iodide (PI) solution (2 µg/mL) for 6 h at 37 °C with gentle shaking and then washed 3-4 times in PBS. Subsequently, immunolabeled tissue samples were cleared.

### Clearing

#### CUBIC

The dissected tissue block was washed 3 times with PBS for half an hour each time at RT to remove PFA. The tissue block was then immersed in CUBIC-L solution [10 wt% N-butyldiethanolamine (TCI #B0725) and 10 wt% Triton X-100 in water], placed on a constant temperature shaker at 37 °C and 80 rpm/min, and shaken for 3 d to decolorize and degrease. Then, the cells were washed three times for 2 h each with PBS at RT. The PBS-washed tissue block was immersed in CUBIC-R solution [45 wt% antipyrine (TCI #D1876) and 30 wt% nicotinamide (TCI #N0078) in water buffered with 0.5% (v/w) N-butyldiethanolamine (pH ~10)] for 1 d at RT. Then, the samples were immersed in new CUBIC-R solution for 1-2 d until clear.

#### SHIELD

The samples were incubated in fresh SHIELD OFF solution at 4 °C with shaking for 2 d. In a 2 mL Eppendorf tube, SHIELD-ON buffer and SHIELD-epoxy solution were mixed at a ratio of 7:1, and the sample was incubated at 37 °C with shaking for 3 h. The samples were then transferred to a new 2 mL Eppendorf tube with an equal volume of fresh SHIELD-ON buffer and incubated at 37 °C with shaking overnight. Then, the samples were incubated in LifeCanvas Passive Clearing Buffer at 45 °C with shaking for 3 d. After clearing was completed, the samples were incubated in PBST (1% Triton X-100 in PBS) overnight at 37 °C to wash out any remaining LifeCanvas Passive Clearing Buffer. Finally, the tissue was incubated in 50% EasyIndex + 50% distilled water with shaking at RT for 3 h, and then the tissues were placed in 100% EasyIndex at RT until transparent.

#### iDISCO+

The sample was pretreated and then dehydrated again using the same dehydration steps and incubated in 66% DCM/33% MeOH for 3 h at RT with shaking. To wash away the MeOH, the mixture was incubated for 15 min twice in 100% DCM with shaking at RT. Finally, the samples were transferred into 100% dibenzylether (DBE) without shaking until transparent.

#### uDISCO

The samples were pretreated following sample pretreatment. After pretreatment or immunolabeling, samples were serially incubated in 5 mL of 30%, 50%, 70%, 80%, 90%, 96%, and 100% tert-butanol at 35 °C for dehydration, and then they were submerged in DCM for 45 min at RT to remove the lipids. They were ultimately treated with BABB-D4 (prepared by mixing BABB (benzyl alcohol + benzyl benzoate 1:2, with diphenyl ether (DPE) at a ratio of 4:1 and adding 0.4% vol DL-alpha-tocopherol (Vitamin E)) for at least 2 h at RT until the samples turned transparent.

### Imaging

This study developed a double ring-modulated Bessel-type light-sheet fluorescence microscope to three-dimensionally image cleared human lymphoma samples. A schematic diagram of its optical path is shown in Figure S1. The system contains three lasers with wavelengths of 488, 532 and 637 nm. The incident light was expanded by three pairs of cylindrical lenses and then focused onto a customized double ring optical mask using a cylindrical lens (CL = 250 mm, Thorlabs). The double ring mask was carefully positioned at the conjugate plane of the back focal plane of the illumination objective using a 75-mm focal length achromatic lens and an infinity-corrected tube lens (TTL100, Thorlabs). After the modulated beam was projected onto the illumination objective (XLFLUOR4X/340, Olympus), a uniform laser sheet with 600-µm interference length (width) and 3.5-µm axial extent (thickness) was generated to illuminate the sample at the focal plane of the orthogonally placed detection objective (UMPLFLN10XW, Olympus). The fluorescence emission from the consecutively excited planes of the sample was then collected by the fluorescence imaging path containing the detection objective, a 200-mm tube lens (ITL200, Thorlabs) and an sCMOS camera (Hamamatsu ORCA-Flash4.0 V2, pixel size 6.5 x 6.5 μm) with an overall magnification of ×11.1, generating the raw image sequences (2048*1024 pixels with 0.59 × 0.59 μm pixel size) at a high speed up to 50 frames per second (fps). The emission light was filtered with bandpass filters (Olympus BA510-550, BA575-625, and Chroma ZET405/488/532/642 for the three laser sources).

The 3D imaging was performed using a motorized sample control system consisting of an x-axis stage (MTS25-Z8, Thorlabs), a y-axis stage (Z825B, Thorlabs) and a z-axis stage (L505, PI). The 600-μm length laser sheet with a 31.5° incident angle optically sectioned an ~300 μm depth of the sample at each time, during which the system consecutively imaged the sample plane by plane along the x- (11 mm dimension with a step size of 2 μm) and y- (5 mm dimension with a step size of 1 mm) axes using a raster scan mode. The z-axis stage was used to repeat such an x-y raster scan with a 250-μm step size (16.7% overlapping depth for poststitching) until the entire depth of the sample (~1000 μm depth) was imaged. All fluorescent channels were collected in sequence in this way.

### Data processing procedure

The acquired image stacks were stitched using ImageJ. Imaris software was used to reconstruct the whole sample and select regions of interest (ROIs) from the acquired images. The spot function within the software was employed to locate and statistically analyze immunofluorescently labeled cells, yielding information such as cell numbers and three-dimensional coordinates. Subsequently, statistical analysis of the three-dimensional cell coordinates was performed using the Pycharm platform (Python 3.10.0), allowing for the calculation of cell density, spatial correlation, and cell conjugation. Cell density was determined by dividing the number of cells of a particular type by the corresponding ROI volume. Cell spatial correlation was determined with a 5-μm interval using the three-dimensional coordinates of cells within a 50-μm radius of the center T-cell to calculate the distribution of B, Treg, M, NE, TAM and other cells. Cell conjugation was defined as the status of closely adjacent cells with distances less than 15 μm. ImageJ software was used to identify cell conjugation between different cell types within the ROIs of different samples.

### Image processing and statistical analysis

Data were processed and analyzed using GraphPad Prism 8.0.2 and SPSS 26.0, statistical analysis was performed using the nonparametric rank sum test (i.e., Kruskal‒Wallis test for multiple samples) and two-way ANOVA, and the graphs were created using Adobe Illustrator CS6. A p value less than 0.05 was considered statistically significant.

## Results

### Selection of clearing methods suitable for human lymphoma tissue

**Figure 1:**
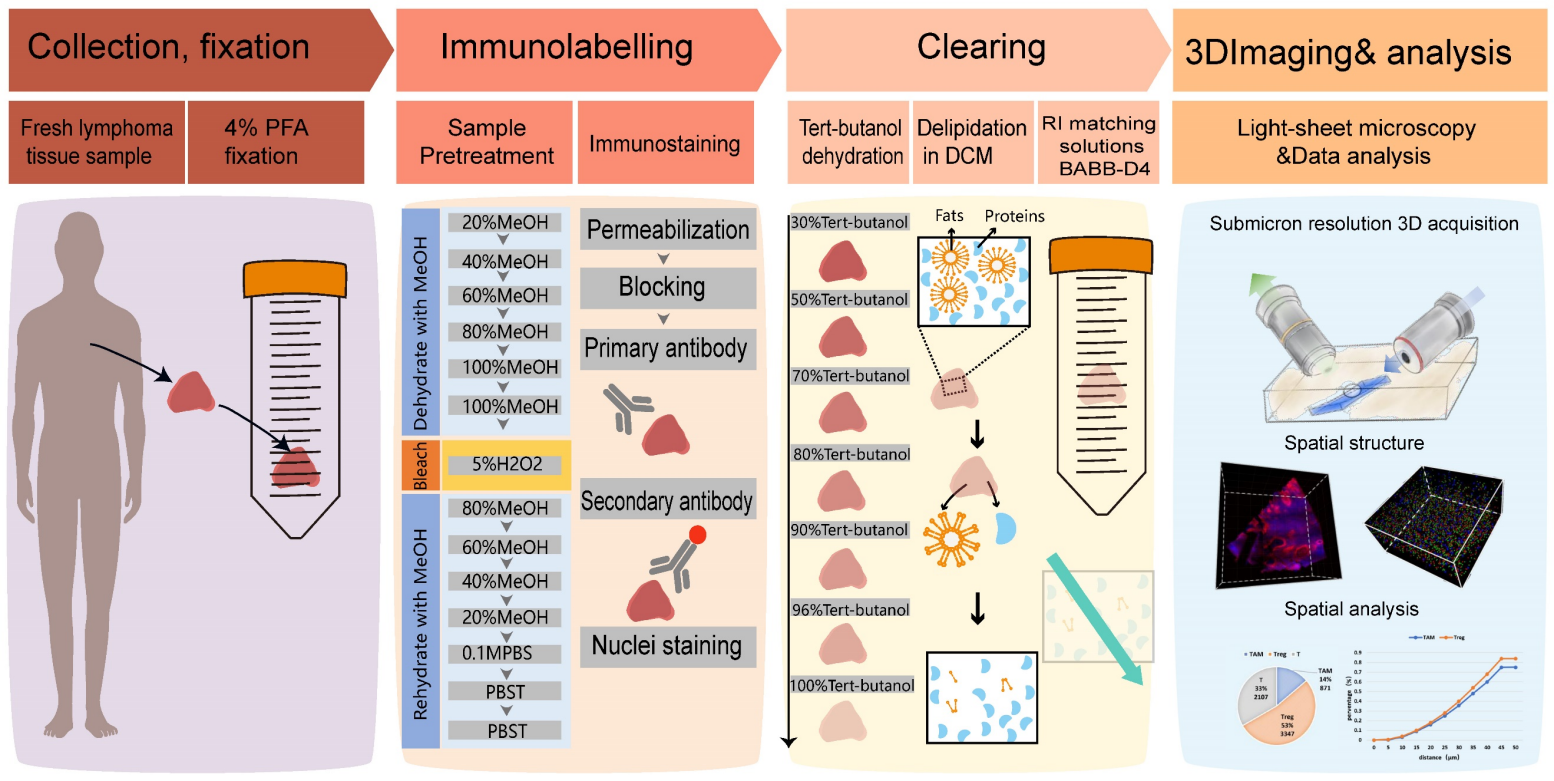
Overview of lymphoma TME 3D imaging and analysis pipeline, which contains four main steps. Step 1: collection of fresh lymphoma tissue samples, which were fixed immediately with 4% PFA and stored at 4℃. Step 2: immune-labeling of samples, wherein samples were first pretreated with methanol series concentration for dehydration, 5% H2O2 for bleaching, and methanol series concentration for rehydration, followed by antibody labeling. Step 3: tissue optical clearing, which included tert-butanol dehydration, DCM delipidation, and matching for BABB-D4 until they became transparent. Step 4: 3D imaging of labelled transparent tissues with a light sheet microscope and image-based pathological analysis of the TME.

**Figure 2:**
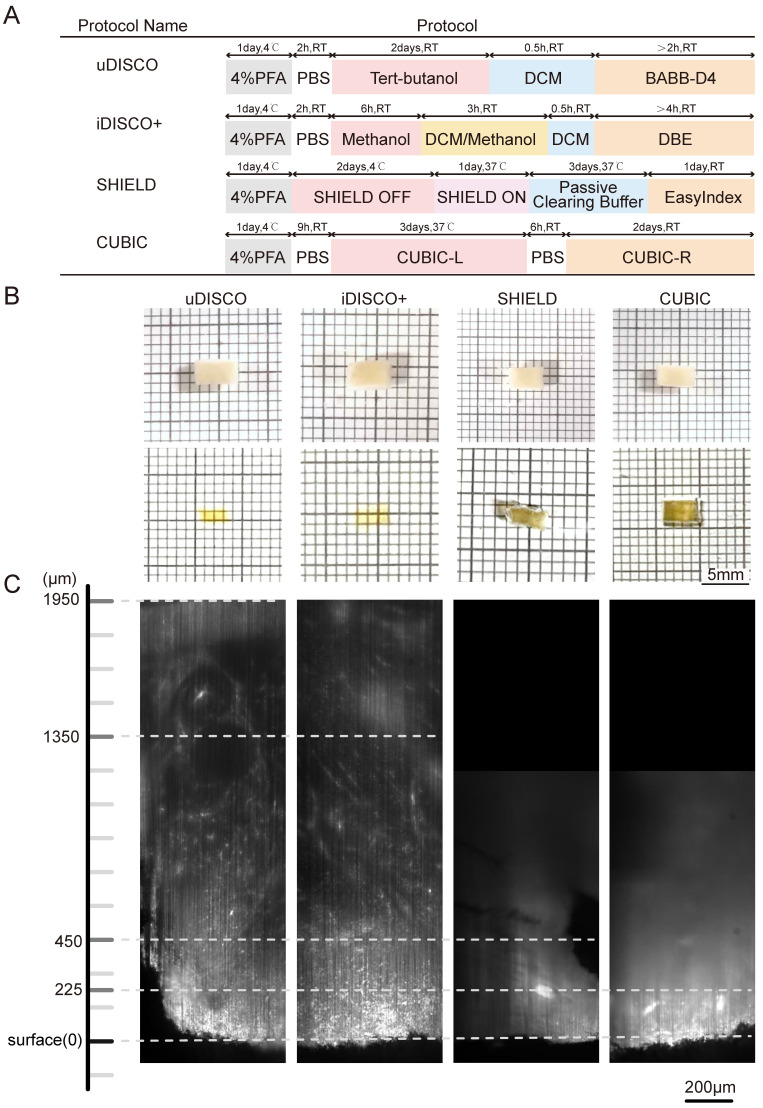
Comparative performances of four tissue clearing methods for human lymph node specimens. (A) Protocol and clearing time of uDISCO, iDISCO+, SHIELD, and CUBIC. (B) Photographs of 1-mm thick human lymph node tissue blocks before and after clearing using uDISCO, iDISCO+, SHIELD and CUBIC. The scale of the grid is 1 mm × 1 mm. (C) Comparison of achievable imaging depth of four clearing methods. Scale bar = 200 μm.

The sample manipulation and imaging workflow is illustrated in Figure 1. Lymphoma tissues were collected, fixed, and immunostained. After dehydration and rehydration, the specimens were cleared by a suitable clearing method until transparent, imaged, and processed for spatial analysis. To select the most appropriate clearing strategy for human lymphoma tissue, we compared four tissue clearing methods, iDISCO+, uDISCO, CUBIC, and SHIELD, which were previously reported to be suited for human specimens. The specimens were cut into a uniform size of 4×3×1 mm^3^ to ensure the consistency of the experiments. The flowchart of the four clearing protocols is shown in Figure 2A. CUBIC and SHIELD required a longer time at 6 to 7 d, while iDISCO+ and uDISCO had the shortest transparency times at approximately 2 and 3 d, respectively.

Lymphoma tissue blocks before and after clearing were compared at the macro- and microscales. The samples treated by uDISCO and iDISCO+ were obviously more transparent than those treated by CUBIC and SHIELD, with only the square lines underneath the uDISCO and iDISCO+ samples being visible (photographs in Figure 2B). Then, the samples were imaged by LSFM using a 532-nm excitation light sheet. The acceptable laser penetration depths for microscopic imaging of the uDISCO, iDISCO+, CUBIC, and SHIELD samples were ~1950 µm, ~1350 µm, ~450 µm, and ~225 µm, respectively (Figure 2C). Given that the preservation of fluorescence signals after uDISCO is greater than that after iDISCO+ 36, we finally chose a potent and label-friendly uDISCO clearing approach to analyze the human lymphoma samples.

### Lymphoma TME antibody panel design and testing compatibility with uDISCO

**Figure 3:**
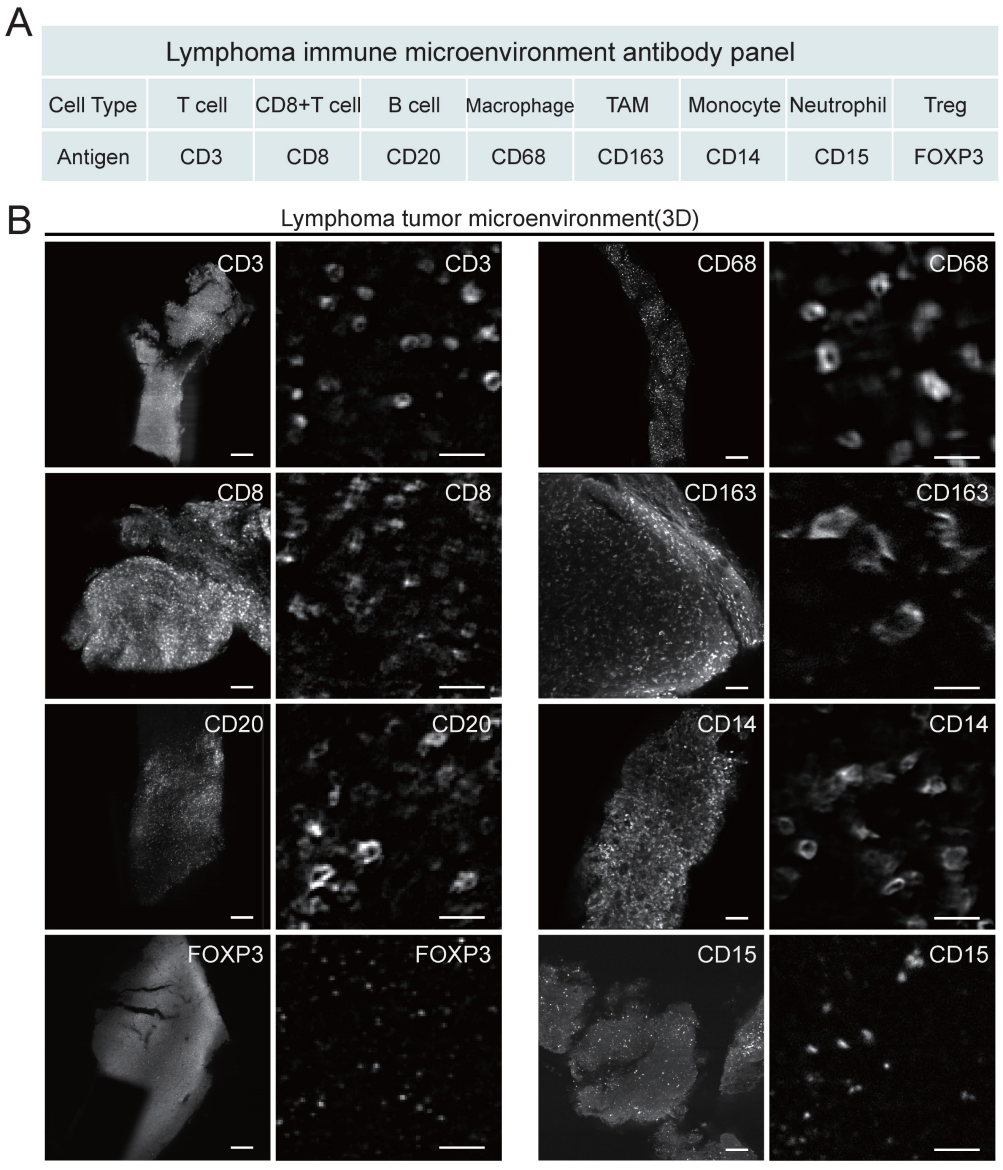
Antibody panel for targeting diverse lymphoma immune cells and LSFM images of each type of labeled immune cells. (A) Screened antibody panel for tagging the interested cell subtypes in the lymphoma TME. (B) The x-y plane images of the cleared lymphoma samples showing the spatial distributions of available 8 antibodies. Scale bars are 200 μm in the left wide-view images, and 20 μm in the right magnified vignettes, respectively.

Considering that the composition of the microenvironment varies in different tumors, we designed a panel specifically for the TME of lymphoma (Figure 3). The panel consisted of primary antibodies against various immune cells, including CD20+ B cells, CD3+ T cells, CD8+ T cells, CD68+ macrophages (Mø), CD163+ tumor-associated macrophages (TAMs), CD14+ monocytes (M), CD15+ neutrophils (NE), FOXP3+ Treg cells, and the proliferation marker Ki67. These immune cells are crucial components of the TME and play an important role in the development and treatment of lymphoma. Considering the compatibility between the uDISCO clearing technique and the immunolabeling antibodies, primary and secondary antibodies were screened first. A total of twenty-six primary antibodies and twelve fluorescent secondary antibodies were validated, and the details of the antibodies are shown in Table S2-3. After validation of the fluorescent secondary antibody specificities and screening for the optimal concentrations (Figure S2), compatibility of the antibodies with uDISCO clearing was tested using 20 μm frozen sections. Antibodies compatible with uDISCO retained a greater fluorescence signal, and 17 out of 26 primary antibodies were verified to be uDISCO-compatible, as shown in Figure S3 and Table S2. Immunofluorescence (IF) and IHC on serial tissue sections were performed to test the compatibility of the antibodies for these two assays. The results revealed high comparability between IF and IHC, which indirectly suggests consistency between 3D immunofluorescence and IHC. (Figure S4).

### Validation of multiplex immunofluorescence labeling

Afterward, we performed multiple immunofluorescence (mIF) validations and found that different excitation wavelengths also have slight impacts on imaging depths. The lymphoma tissue blocks were labeled with primary antibodies against CD68, FOXP3, and CD8, followed by incubation with Alexa Fluor 488, Alexa Fluor 555 and Alexa Fluor 647 fluorescent secondary antibodies. The fluorescent secondary antibodies were excited at wavelengths of 488, 532, and 637 nm during LSFM imaging. The depths of light penetration for the 488, 532, and 637 nm wavelengths were ~660 μm, 780 μm, and 900 μm, respectively (Figure S5). This finding is consistent with the previous knowledge that excitation light with a longer wavelength can penetrate tissue more effectively 24, 37. To verify whether 3D immunolabeling following uDISCO clearing lymph nodes were still amenable to classical immunohistological analysis, we performed H&E and IHC staining separately on sections of LSFM imaged tissue. The results showed that immunolabeling and clearing did not affect the following H&E and IHC staining at all. So, it is feasible to apply LSFM 3D imaging and classical immunohistological analysis on the same tissues (Figure S6). In addition, the 3D visualization of macrophage, Treg and CD8+ T-cell structures in lymphoma tissue was accomplished, as shown in Supplementary Movie 1. uDISCO in combination with mIF labeling and LSFM imaging techniques was then applied to study the lymphoma tissue.

### LSFM imaging of the TME in human lymphoma tissues

**Figure 4:**
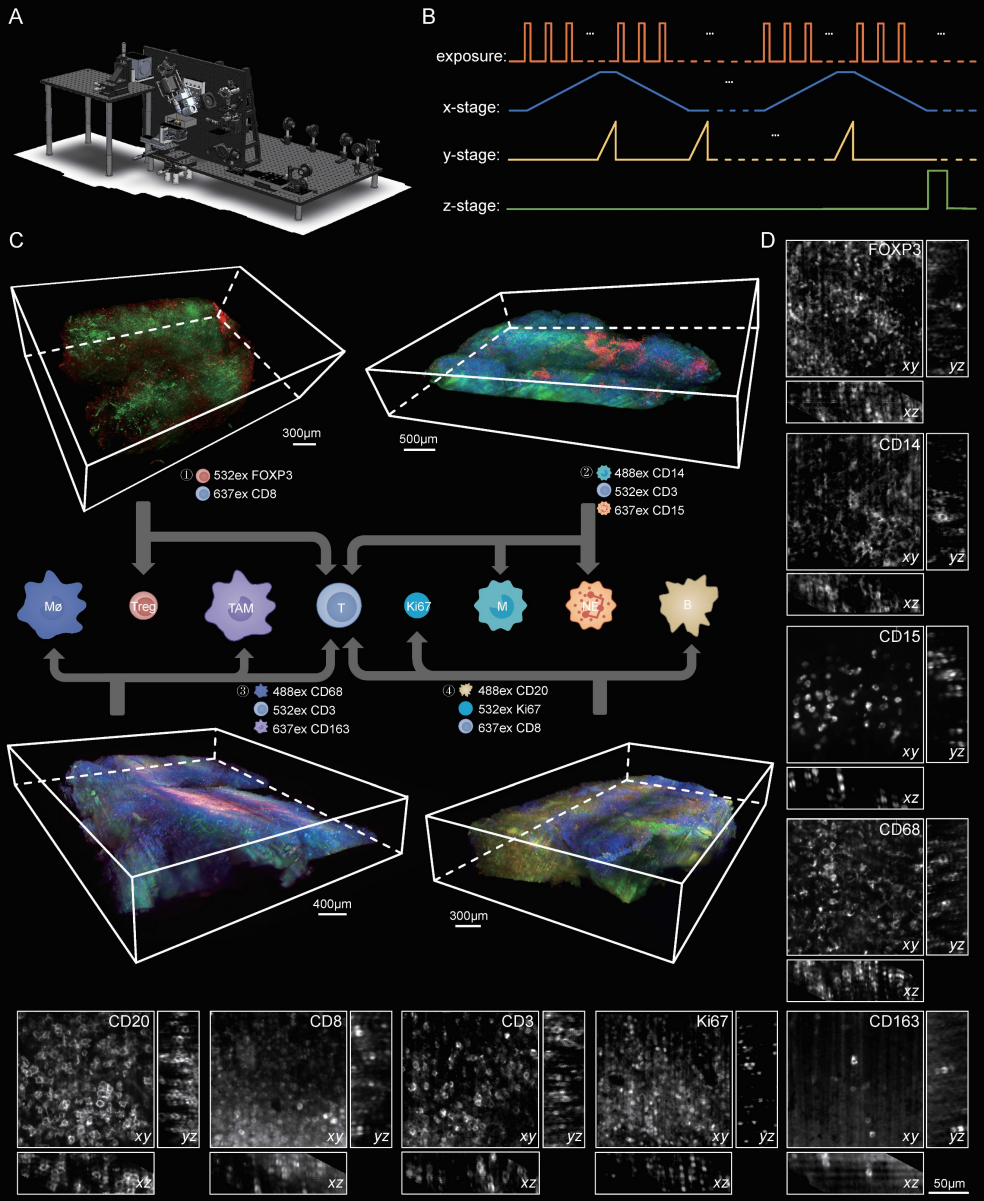
3D visualization of the immune TME in lymphoma at single-cell resolution. (A) The schematic of our home-built light-sheet fluorescence microscope suited for high-speed imaging of cleared thick samples. (B) Control diagram of the camera and motorized stages of the microscope during imaging (C) Volume renderings of the human lymphoma immune TME. A lymphoma specimen was divided into four parts with each part labelled with different cell subtypes. ① Tregs and T cells: CD8, red; FOXP3, green. Scale bar = 300 μm. ②Monocytes, neutrophils and T cells: CD14, blue; CD15, red; CD3, green. Scale bar = 500 μm. ③ Macrophages, TAMs and T cells: CD68, blue; CD163, red; CD3, green. Scale bar = 400 μm. ④ B cells, T cells and Ki67: CD20, blue; CD8, red; Ki67, green. Scale bar = 300 μm. (D) Close-up xy and yz planes of diverse antibodies-labeled cell subtypes (CD3, CD8, CD20, CD68, CD163, CD14, CD15, FOXP3 and Ki67). Scale bars = 50 μm in all images.

To provide a 3D visualization of the entire lymphoma TME, we dissected the same patient lymphoma specimen into four blocks, each 500 μm thick, and then performed multicolor staining on each tissue block. The corresponding antibody staining combinations for each tissue block are documented in Table S4. After multiplex labeling and clearing procedures, the samples were transparent, specifically tagged, and ready for LSFM imaging. The diagram of our self-developed Bessel-type LSFM is shown in Figure 4A. Figure 4B shows the optoelectronic control diagram of the system. LSFM image data were imported into Imaris software, and a 3D spatial presentation was established (Figure 4C). We used 3D visualization to show the TME cytoarchitectures of immune cells/Ki67 in Part 1, T cells/macrophages/TAMs in Part 2, neutrophils/monocytes/T cells in Part 3, and T cells/Treg cells in Part 4. These results together provided a 3D panorama of the lymphoma immune TME. We further selected several ROIs from the panoramic views to show the high-resolution vignettes of the xy, yz and xz planes for each antibody marker (Figure 4D). The single cells could be clearly discerned for each subtype of labeled cells.

### 3D cell quantification and spatial correlation analysis

The TME of lymphoma is highly heterogeneous, and the number and function of immune cells in the TME can significantly affect patient outcomes 11, 38-39. To investigate the differences between the TMEs of different lymphomas, we collected patient samples from three types of diseases: RLN, DLBCL and AITL (Table S1). Photographs of these three diseased tissues before and after clearing are shown in Figure S6, and a 3D visualization is shown in Figure S8. The quantity and spatial distribution of immune cells in the TME of these three diseases were compared.

To ensure uniformity, we divided each patient sample into four evenly sized tissue blocks and treated them with the same staining protocol (Table S4). 3D visualization of the 12 tissue blocks before and after clearing was performed, as shown in Figures S7. Next, LSFM image data of the 12 tissue blocks were imported into Imaris for ROI selection and 3D reconstruction. For each tissue block, ten ROIs were selected for data analysis, and the spot function of Imaris software was used to localize immunofluorescence-labeled cells and obtain the statistical data. The procedure for cell counting with Spot is illustrated in Figure S9. The cells were automatically segmented, and their 3D coordinate information was obtained to calculate the cell numbers and intercellular distances.

**Figure 5:**
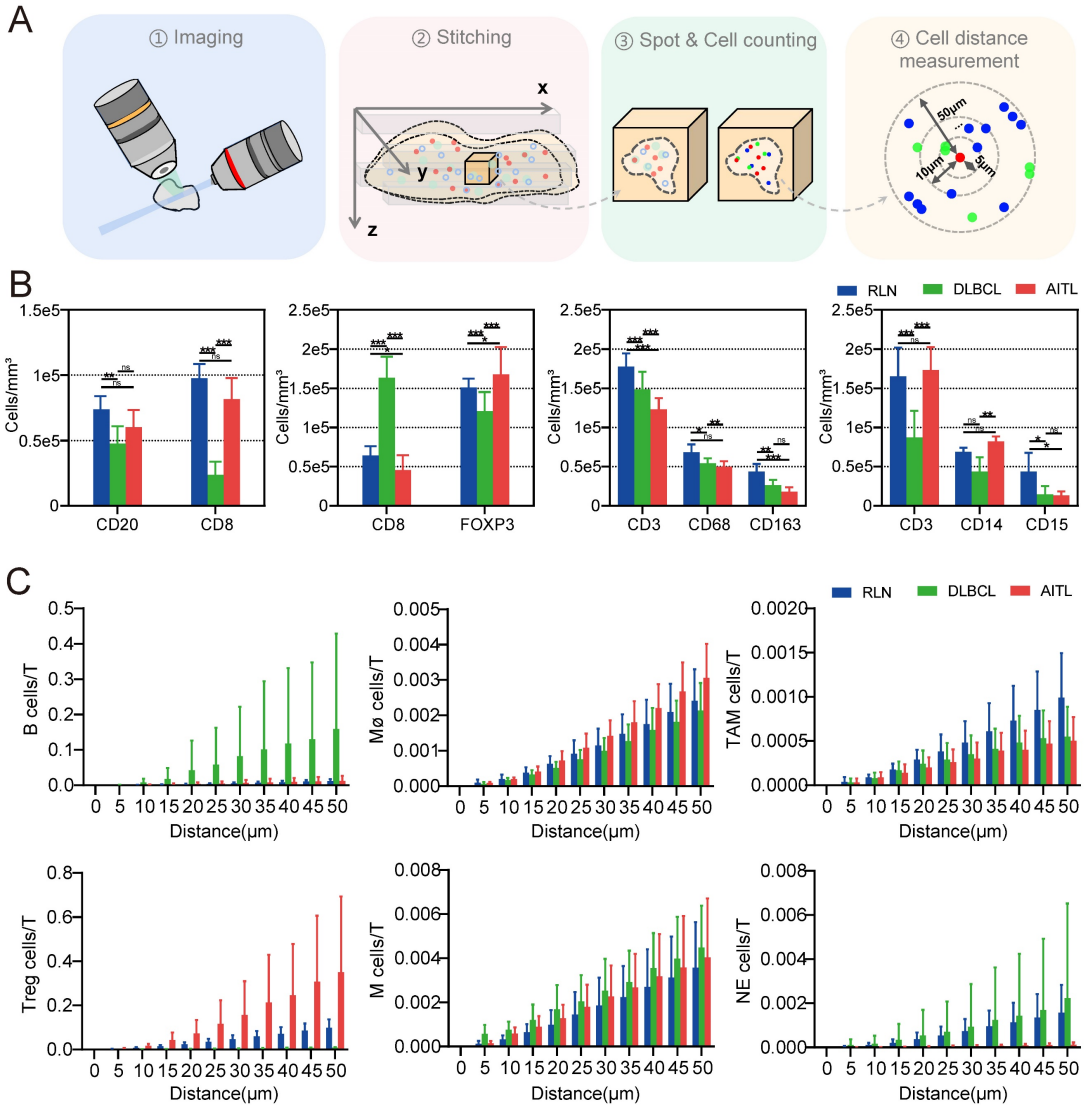
LSFM image-based data processing flow for analyzing the spatial correlations in the lymphoma TMEs. (A) Post analysis pipeline of the multiplexed LSFM images. (B) The density (cells per mm^3^) of immune cell types in lymph nodes from three donors with RLN, DLBCL and AITL. i) B cells and T cells: CD20, blue; CD8, red. ii) Tregs and T cells: FOXP3, green; CD8, red. iii) Macrophages, TAMs and T cells: CD68, blue; CD163, red; CD3, green. iv) Monocytes, neutrophils and T cells: CD14, blue; CD15, red; CD3, green. (C) The number of T cells around each immune cell was plotted as a function of the radial distance from the immune cell. Different color bars represent different diseases with blue for RLN, green for DLBCL, and red for AITL.

We quantified the densities of CD3-, CD8-, CD20-, CD68-, CD163-, CD14-, CD15-, and FOXP3-positive cells (Figure 5B) and found that these immune cells were present in all three types of diseased tissues. Interestingly, the number of T cells in RLN, DLBCL and AITL was significantly higher than that of other immune cells. It is well known that T cells are the most important cytotoxic cells in the TME. Immune cells within the TME can affect patient prognosis by promoting or suppressing T-cell function. Furthermore, the numbers of various types of immune cells surrounding T cells within 50 μm were counted (Figure 5C). Our results showed that the number of B cells around T cells was significantly higher in DLBCL tissue than in RLN and AITL tissue. This finding is consistent with the fact that DLBCL is a B-cell-dominated disease. In AITL tissue, the number of Treg cells and macrophage cells around T cells was higher than that in RLN and DLBCL tissues, but the number of NE cells surrounding T cells was lower than that in RLN and DLBCL tissues. This AITL patient had more immunosuppressive cells, such as Tregs and macrophages, around T cells and fewer NE cells, indicating that the T cells may be in an immunosuppressed state.

### Immune cell conjugation within the TME of lymphoma

**Figure 6:**
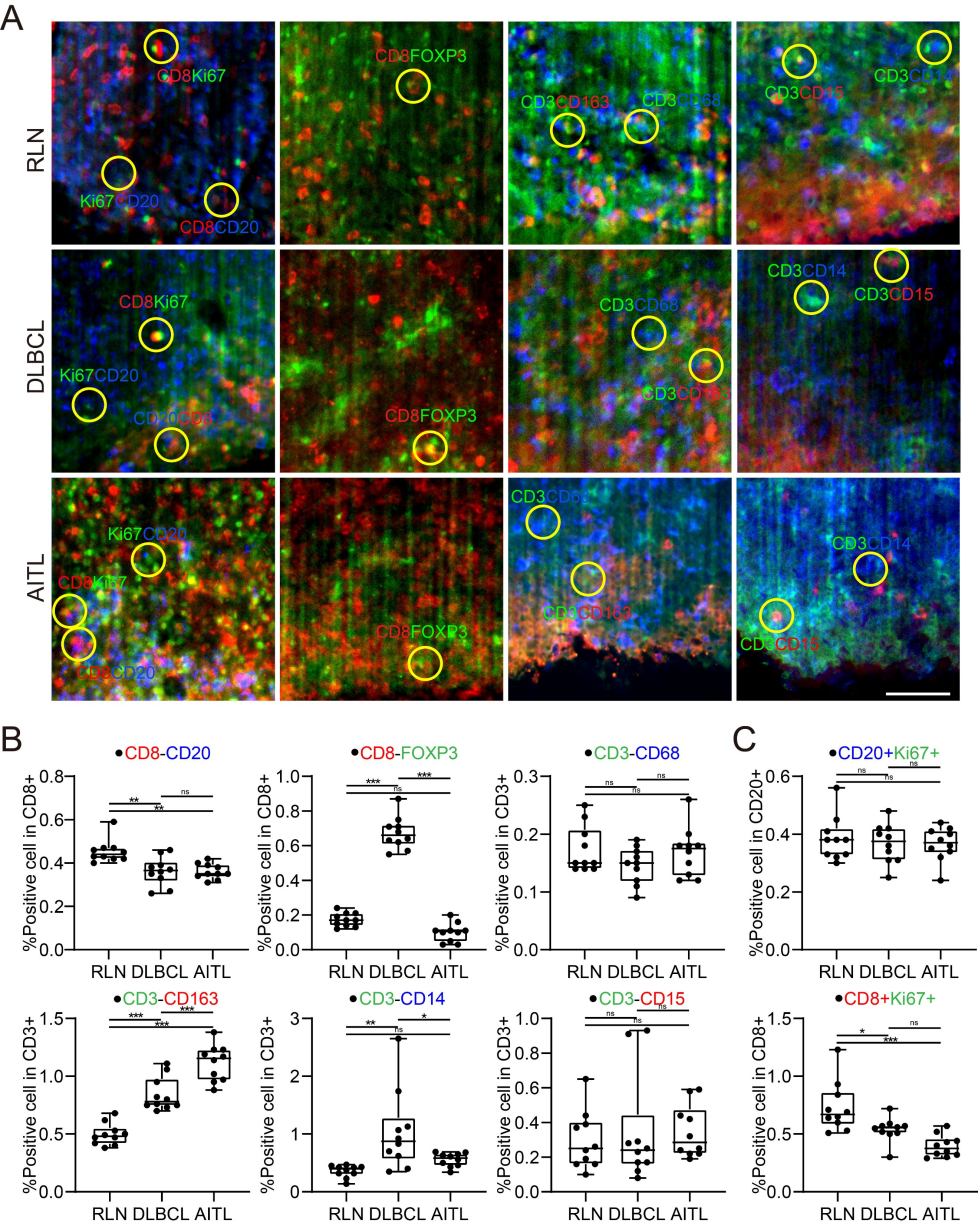
Spatial analysis of the colocalizations among diverse immune cells in the lymphoma TME. (A) Microenvironment images directly showing the colocalization among the specific immune cells in the RLN, DLBCL and AITL. The yellow circles indicate the cell colocalizations including CD8 (red) and CD20 (blue); CD8 (red) and FOXP3 (green); CD3 (green) and CD68 (blue); CD3 (green) and CD163 (red); CD3 (green) and CD14 (blue); and CD3 (green) and CD15 (red). (B) The boxplots revealing the differences of immune cell colocalizations in RLN, DLBCL, and AITL. (C) The comparative proliferative capacities of B cells and T cells in RLN, DLBCL, and AITL.

It was reported that patient prognosis could be impacted by the interaction between T cells and other immune cells within the lymphoma TME. In this study, we examined the conjugation of different immune cells with T cells in RLN, DLBCL and AITL tissues. At a distance of less than 15 μm, point objects of different cell types were considered conjugate 40. We performed conjugation analysis of B/CD8+ T, CD8+ T/Treg, T/Mø, T/TAM, T/M, and T/NE cells. The direct conjugations of CD8/CD20, CD8/FOXP3, CD3/CD68, CD3/CD163, CD3/CD14, and CD3/CD15 are shown in Figure 6A. Statistical analysis of CD3-CD163 conjugations showed significant differences among RLN, DLBCL and AITL tissues (Figure 6B). In the three cases of this study, the number of CD3-CD163 conjugations in AITL was higher than that in DLBCL and RLN tissues. While these TAMs are immunosuppressive cells, the excessive conjugations between them indicates that T cells may be in an immunosuppressive state. CD3-CD68 and CD3-CD15 conjugation numbers were not significantly different among the cancer tissues. Furthermore, we examined the proliferative capacity of B cells and T cells by counting the number of Ki67+ CD20+ B cells and KI67+ CD8+ T cells (Figure 6C). The results revealed that the ratio of Ki67+ cells to CD20+ cells was not significantly different among RLN, DLBCL, and AITL tissues, while the ratio of Ki67+ cells to CD8+ cells was significantly different among the cancer tissues (p = 0.0001). It is also noted that T cells exhibited the strongest proliferative capacity in the RLN sample. Altogether, these results demonstrated that advanced LSFM imaging of cleared mIF tissues could provide efficient 3D visualization of the lymphoma TME and further enable statistical studies revealing the spatial correlations between immune cells *in situ*.

## Discussion

Even though there are many tissue clearing techniques, the type of tissue clearing method especially suited for a specific tissue has not yet been well studied. Each tissue clearing technique possesses its own advantages and disadvantages. The choice of tissue clearing techniques for diverse species and tissue sites should be adaptive to the specific research purpose 41. In this study, we compared iDISCO+ 42, uDISCO 43, CUBIC 44, and SHIELD 45 on millimeter-sized human lymphoma tissue samples to determine the most appropriate method for the experiment. After carefully leveraging the transparency of the sample, compatibility with immunolabeling, and preservation of fluorescence, we finally chose a modified uDISCO clearing method for the treatment of lymphoma tissue samples. Moreover, the tert-butanol dehydration and BABB-D4 clearing reagents we used in this study only lead to mild and isotropic tissue shrinkage in three dimensions, allowing the clarified tissues to be spatially analyzed in near native states 43, 46.

Antibody-specific immunolabeling is the technique of choice for visualizing diverse cell subtypes in dissected tumor tissues and needs to be jointly optimized with clearing techniques during 3D tissue clearing and labeling 47. In this work, we assessed the appropriate antibody concentrations on 20-µm-thick frozen sections and the optimal antibody incubation times on 1-mm-thick human lymphoma tissues. Finally, we screened the antibody panel suited for labeling the lymphoma TME. It should be noted that the labeling intensity and SNR of antibodies can vary with different antibodies due to various factors, such as manufacturing source and production batch.

The TME has been implicated in the development, invasion, and metastasis of lymphoma as well as other tumors 48-50. However, the spatial properties of the TME in lymphoma are not well known due to the difficulty in visualizing the diverse cell subtypes across the entire tissue. The advent of LSFM paves the way for *intoto* 3D imaging of clarified tissue at a speed far superior to classic confocal microscopy implementations 51-52. In this study, we accomplished the first 3D representation of the spatial structures of the lymphoma TME, including various cell types, such as B, T, Mø, TAM, M, NE, and Treg cells. Although only up to three markers can be used to stain one sample due to the limitations on the source of antibody species, we used multiple tissue blocks from the same dissected tissue and the same patient to visualize the high content of cell subtypes in the TME. We also envision that along with the further addition of the tyramide-signal-amplification (TSA) technique, we can overcome the antibody species limitation and achieve multiplex imaging of the same sample with over 6 types of labeled cells.

## Conclusion

In summary, we developed a method combining tissue clearing, mIF labeling and LSFM imaging techniques to realize 3D reconstruction and quantitative analysis of the intact TME in fixed human lymphoma tissues. This work represents the first known study in this field where the lymphoma TME is revealed using advanced approaches. We achieved single-cell resolution across thick lymphoma specimens with depths up to ~1000 µm, expanding the volume size over 100 times when compared to traditional 4-5 µm immunostained slices. In addition, we comprehensively analyzed the spatial information of the lymphoma TME to realize the localization, quantification, cell interactions and intercellular distance profiling of diverse immune cells and tumor cells within the TME. Our work suggests a new paradigm shift for *in situ* pathological studies of the TME in lymphoma and potentially other tumors, and hopefully, it will continue to provide new insights into tumorigenesis and cancer immunotherapy.

## Supplementary Material

Supplementary figures and tables.

Supplementary video.

## Abbreviations

TME: tumor microenvironment
RLN: reactive lymphoid hyperplasia
DLBCL: diffuse large B cell lymphoma
AITL: angioimmunoblastic T-cell lymphoma
LSFM: light sheet fluorescence microscopy
iDISCO+: immunolabeling-enabled three-dimensional imaging of solvent-cleared organs
uDISCO: ultimate dimensional imaging of solvent-cleared organs
CUBIC: clear, unobstructed brain imaging cocktails and computational analysis
SHIELD: stabilization to harsh conditions via intramolecular epoxide linkages to prevent degradation
mIF: multiplex immunofluorescence
Mø: macrophages
TAM: tumor-associated macrophages
M: monocytes
NE: neutrophils
