# Supplementary material for: Efficient 3D imaging and pathological analysis of the human lymphoma tumor microenvironment using light-sheet immunofluorescence microscopy
Source: Theranostics. 2024 Jan 1;14(1):406–19. doi: 10.7150/thno.86221 (PMC10750216; doi:10.7150/thno.86221)
Supplement: Supplementary file 1 — Supplementary figures and tables. [file thnov14p0406s1.pdf]

Supplementary information

Supplementary Tables

Table S1. Human lymph node sample used in this work.

Patient	Collection site	Age	Gender	Diagnosis	Fixative	Fixation
Patient 1	Lymph node	20	female	RLN	4 % paraformaldehyde	7 months
Patient 2	Lymph node	61	male	DLBCL	4 % paraformaldehyde	7 months
Patient 3	Lymph node	42	female	AITL	4 % paraformaldehyde	2 months

Abbreviations: RLN: reactive lymphoid hyperplasia; DLBCL: diffuse large B cell lymphoma; AITL: angioimmunoblastic T-cell lymphoma.

Table S2. Antibodies labeling tested.

Component	Marker	Species	Clone	Dilution	Catalog	Performance
B cell	CD19	Rb	EPR5906	1:50	ab134114	Good
	CD19	Mu	FMC63	1:20	NBP2-52716	Good
	CD20	Rb	EP459Y	1:20	ab78237	Good
T cell	CD3	Rat	CD3-12	1:50	ab11089	Good
	CD3	Rat	CD3-12	1:50	MCA1477T	Good
	CD3	Rat	CD3-12	1:50	GTX42110	Good
	CD8	Mu	4B11	1:50	MCA1817T	Good
	CD8	Mu	C8/144B	1:50	ab17147	Good
	CD4	Rb	EPR6855	1/100	ab133616	Good
	CD4	Rb	Polyclonal	1:50	NBP1-19371SS	Good
Immune cell	FOXP3	Rat	PCH101	1:50	14-4776-80	Good
	FOXP3	Mu	206D	1:50	Cat#320102	Good
	CD68	Rb	EPR20545	1/100	ab213363	Good
	CD68	Mu	KP1	1:50	ab955	Good
	CD163	Mu	EDHu-1	1:50	NB110-40686	Good
	CD14	Rb	SC69-02	1:50	NBP2-67630	Good
	CD15	Mu	MMA	1/100	ab17080	Good
Cell proliferation	Ki67	Rb	SP6	1/200	ab16667	Good
	Ki67	Rat	SolA15	1/100	14-5698-82	Good

Table S3. Fluorescent secondary antibodies.

Fluorescent secondary antibodies	Company	Catalog	Final antibody concentrations
Goat anti-Rabbit IgG (H+L) Highly Cross-Adsorbed Secondary Antibody, Alexa Fluor Plus 488	Thermo Fisher	A32731	10 μg/mL
Goat anti-Rabbit IgG (H+L) Highly Cross-Adsorbed Secondary Antibody, Alexa Fluor Plus 647	Thermo Fisher	A32733TR	10 μg/mL
Goat anti-Mouse IgG (H+L) Highly Cross-Adsorbed Secondary Antibody, Alexa Fluor Plus 488	Thermo Fisher	A32723TR	10 μg/mL
Goat anti-Mouse IgG (H+L) Highly Cross-Adsorbed Secondary Antibody, Alexa Fluor Plus 647	Thermo Fisher	A32728TR	10 μg/mL
Goat anti-Rat IgG (H+L) Highly Cross-Adsorbed Secondary Antibody, Alexa Fluor Plus 594	Thermo Fisher	A48264	10 μg/mL
Goat anti-Rat IgG (H+L) Highly Cross-Adsorbed Secondary Antibody, Alexa Fluo Plus 647	Thermo Fisher	A48265	10 μg/mL
Goat anti-Rat IgG (H+L) Highly Cross-Adsorbed Secondary Antibody, Alexa Fluo Plus 555	Thermo Fisher	A48263	10 μg/mL
Goat Anti-Mouse IgG H&L (Alexa Fluor 647)	abcam	ab150115	5μg/mL
Goat Anti-Rabbit IgG H&L (Alexa Fluor 488)	abcam	ab150077	5μg/mL
Goat Anti-Rat IgG H&L (Alexa Fluor 594)	abcam	ab150160	5μg/mL
Alexa Fluor 594 AffiniPure Donkey Anti-Rat IgG (H+L)	Jackson ImmunoResearch	712-585-150	10 μg/mL
Alexa Fluor 488 AffiniPure Donkey Anti-Rabbit IgG (H+L)	Jackson ImmunoResearch	711-545-152	10 μg/mL

Table S4. Staining protocol for TME of lymphoma tissue blocks.

TME Panel	Primary antibody	Fluorescent secondary antibody
Panel 1	Mouse anti-human CD8	Goat Anti-Mouse IgG （H+L) Alexa Fluor 647
	Rat anti- human FOXP3	Goat Anti-Rat IgG （H+L) Alexa Fluor 555
Panel 2	Rat anti- human CD3	Goat Anti-Rat IgG （H+L) Alexa Fluor 555
	Rabbit anti- human CD14	Goat Anti- Rabbit IgG （H+L) Alexa Fluor 488
	Mouse anti-human CD15	Goat Anti-Mouse IgG （H+L) Alexa Fluor 647
Panel 3	Rat anti- human CD3	Goat Anti-Rat IgG （H+L) Alexa Fluor 555
	Rabbit anti- human CD68	Goat Anti- Rabbit IgG （H+L) Alexa Fluor 488
	Mouse anti-human CD163	Goat Anti-Mouse IgG （H+L) Alexa Fluor 647
Panel 4	Mouse anti-human CD8	Goat Anti-Mouse IgG （H+L) Alexa Fluor 647
	Rabbit anti- human CD20	Goat Anti- Rabbit IgG （H+L) Alexa Fluor 488
	Rat anti- human Ki67	Goat Anti-Rat IgG （H+L) Alexa Fluor 555

Supplementary figures

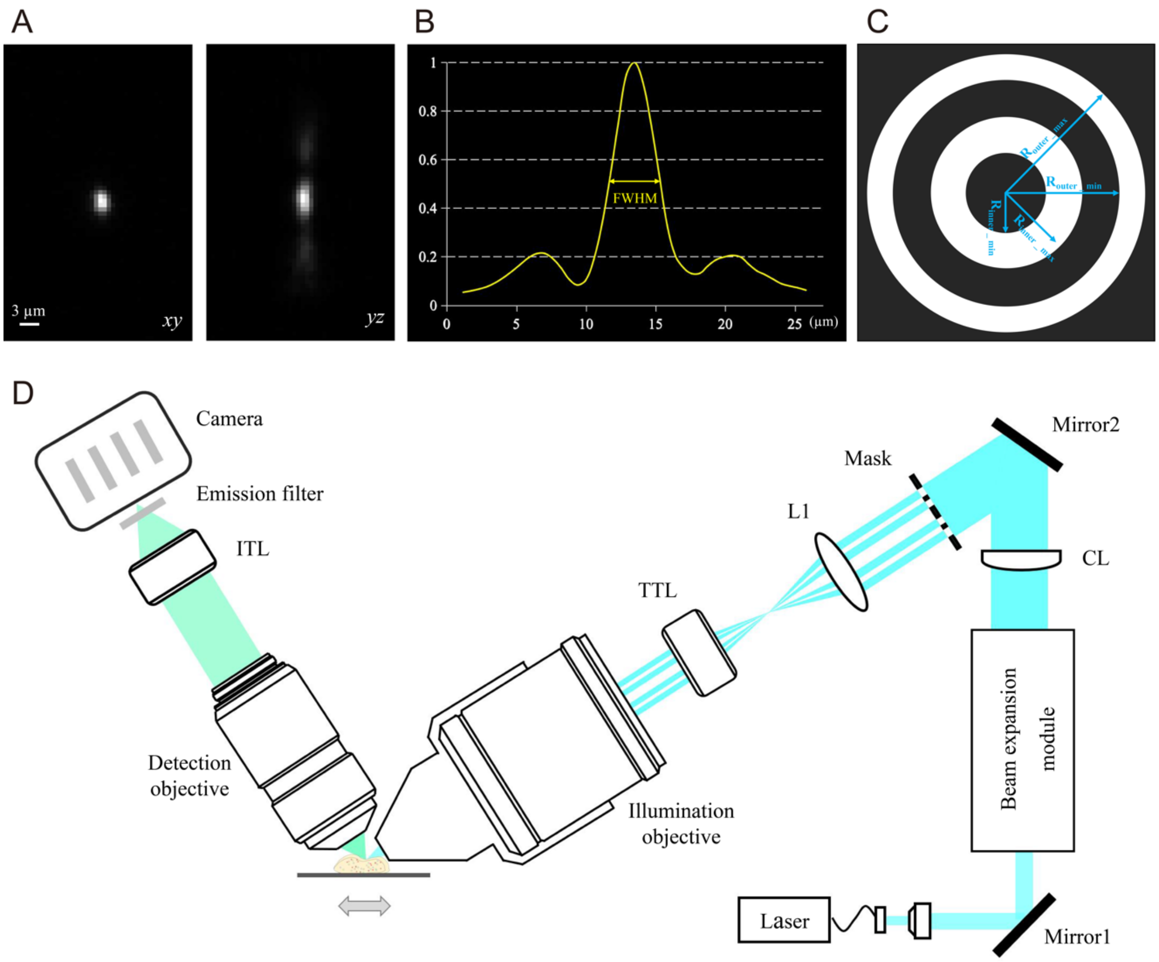

Figure S1. The implementation of double-ring-modulated Bessel light sheet microscopy. (A-B) Shows the point spread function (PSF) of the whole system. A non-diffraction laser-sheet measured ~3.5-um in thickness and <25% sidelobes was generated using the ring modulation mask shown in (C). The radii of the mask, R_{outer_max}, R_{outer_min}, R_{inner_max} and R_{inner_min}, are 2.541, 2.502, 1.509 and 0.397 mm, respectively. (D) Schematic illustrating the optical path of our double-ring selective plane illumination microscope.

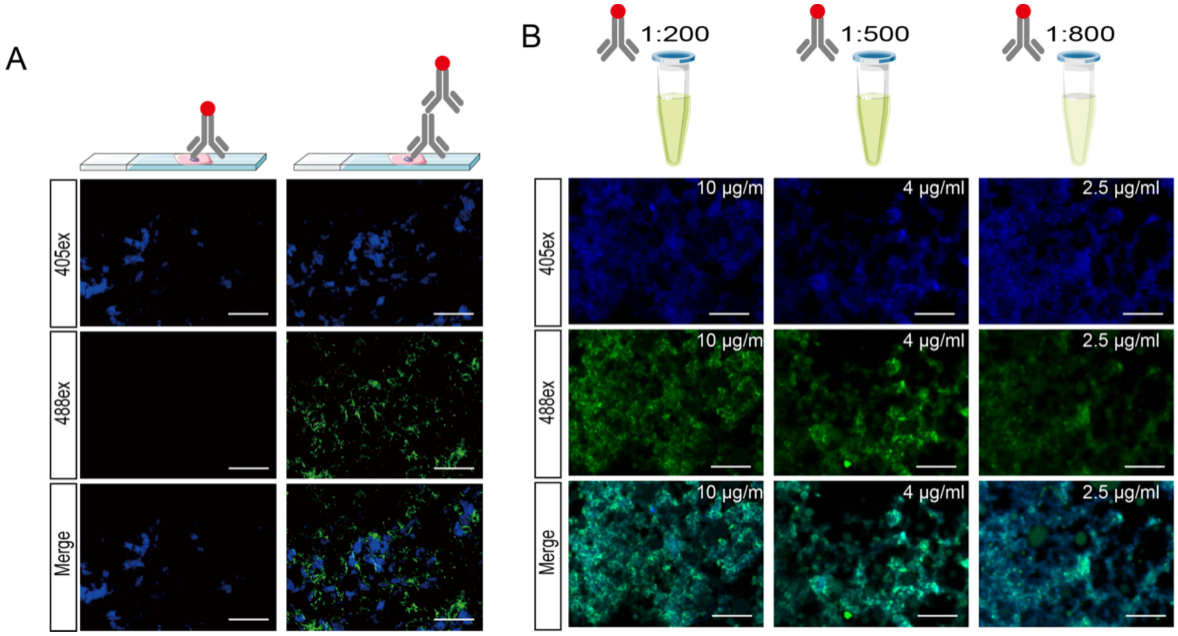

Figure S2. Validation of the specificity of diverse fluorescent secondary antibodies and screening for the optimal concentration, scale bars = 20 μm. (A) Fluorescent secondary antibody specificity verification. (B) Screening for optimal concentration of fluorescent secondary antibody, scale bars = 20 μm.

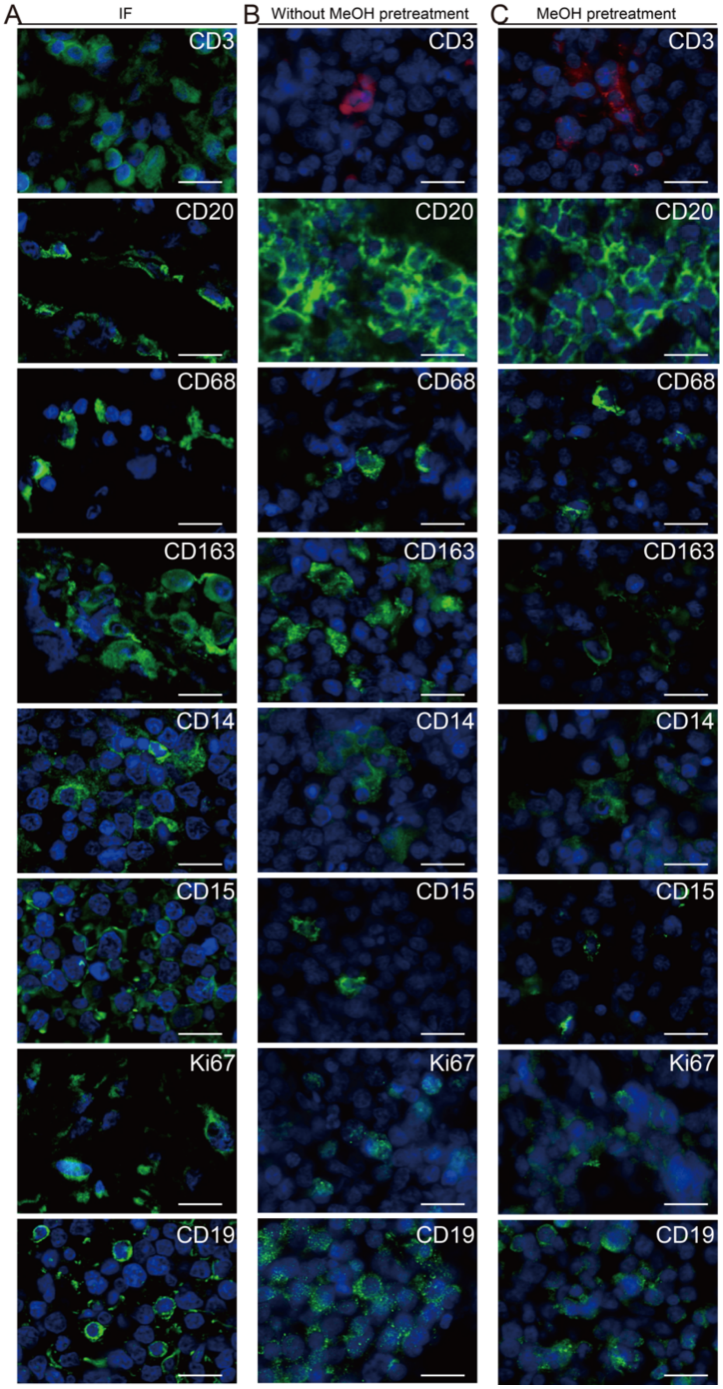

Figure S3. Antibody Validation. We test whether the antibodies can be fluorescently labeled onto methanol-pretreated tissues to screen the candidates suited for uDISCO clearing protocol. (A) Antibodies compatible with immuno-fluorescence labeling, scale bars = 20 μm. (B) Immuno-fluorescence images of tissue sections without methanol pretreatment, scale bars = 20 μm. (C) Immuno-fluorescence images of methanol pretreated tissue sections, scale bars = 20 μm

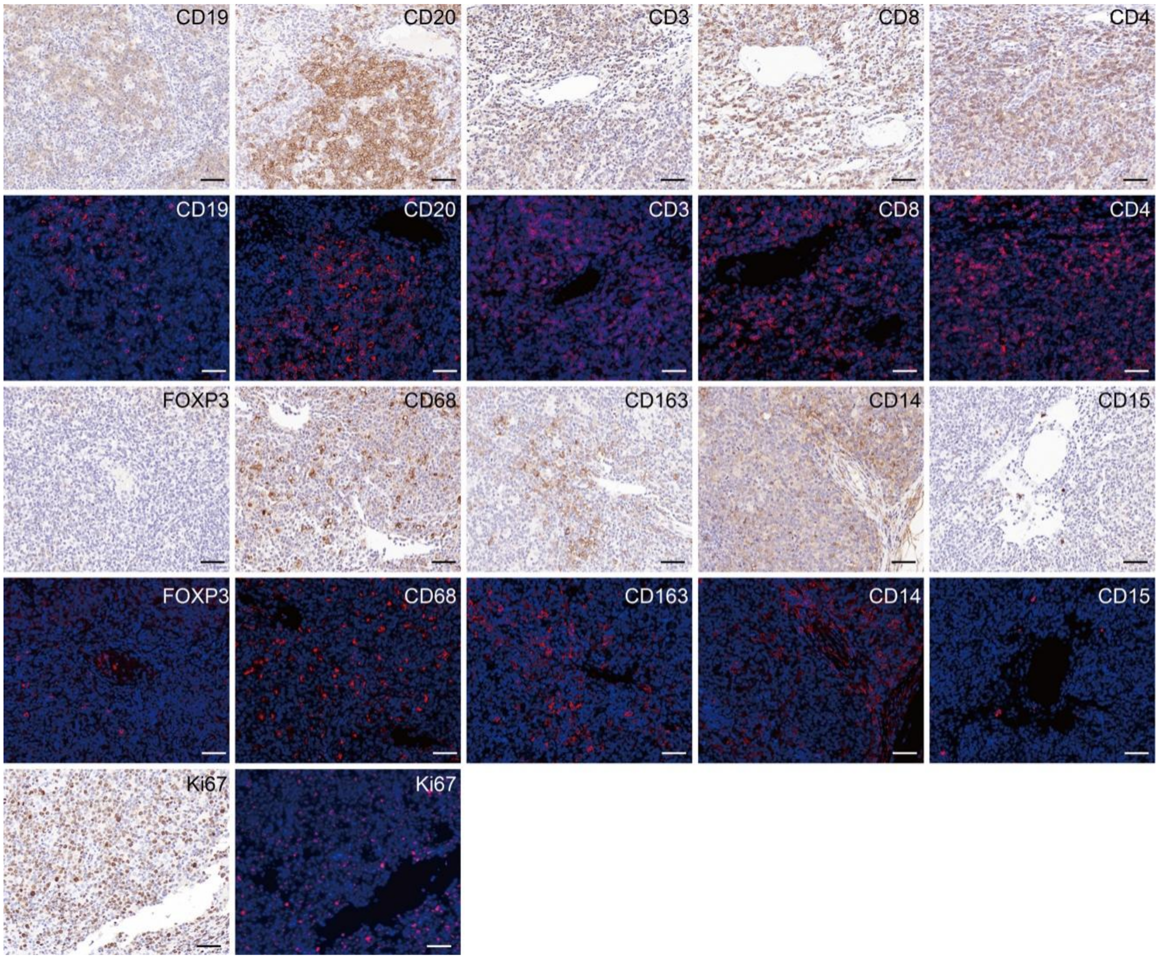

Figure S4. Microscopy images of diverse antibodies comparing the immuno-fluorescence signals and IHC signals in serial tissue sections, scale bars = 50 μm.

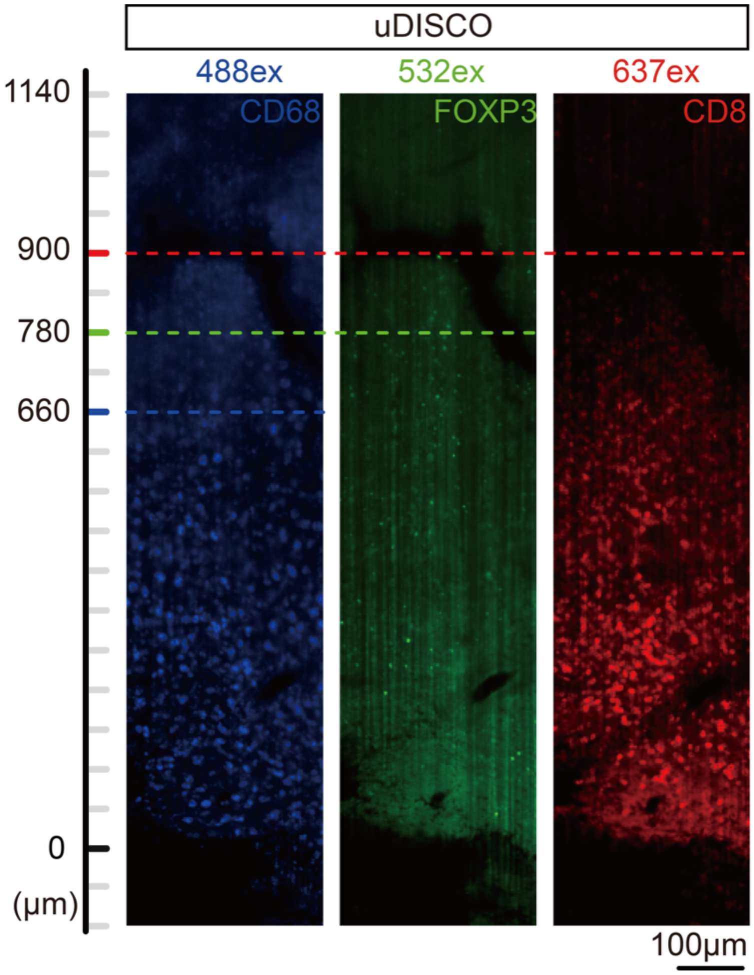

Figure S5. Effective imaging depths of the same cleared lymphoma tissue with using 488-nm (left), 532-nm (middle) and 637-nm (right) excitation wavelengths. Scale bar = 100 μm.

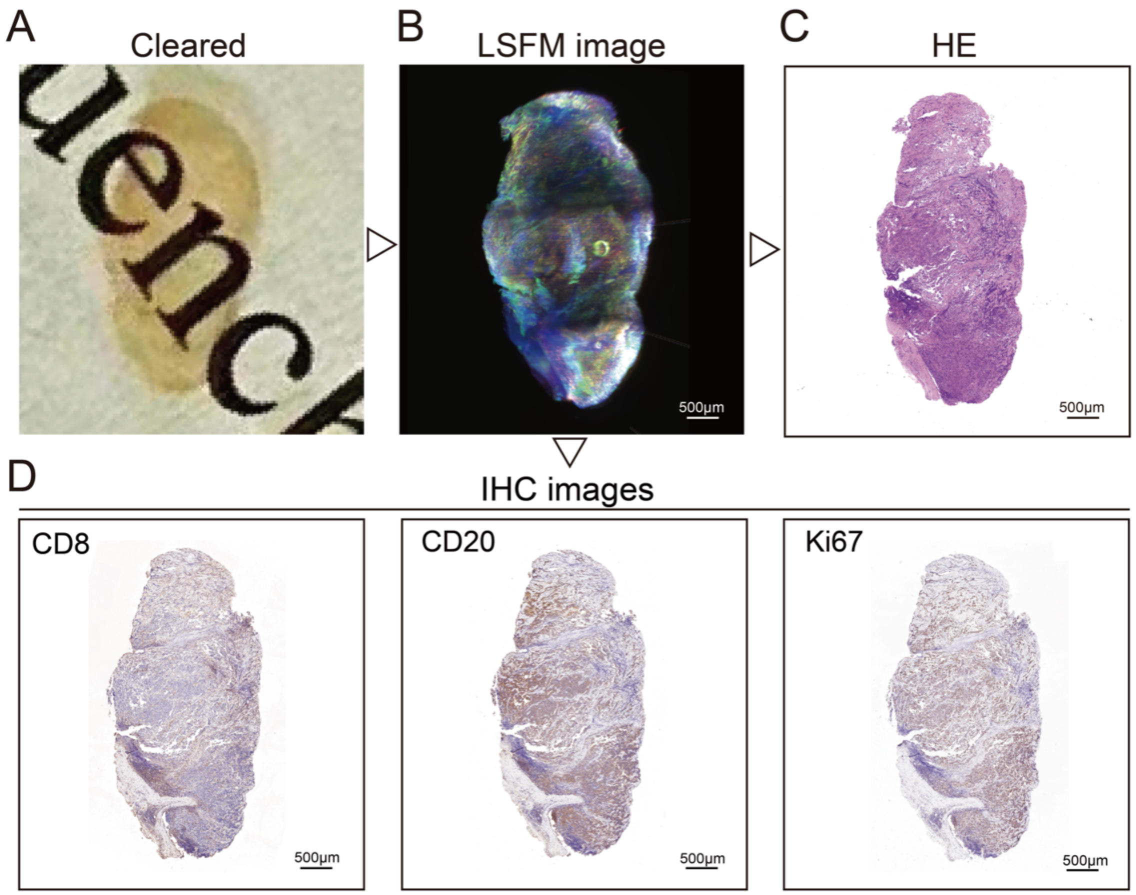

Figure S6. Compatibility testing of 3D immunolabeling following uDISCO clearing (A-B) and H&E (C) as well as IHC staining (D).

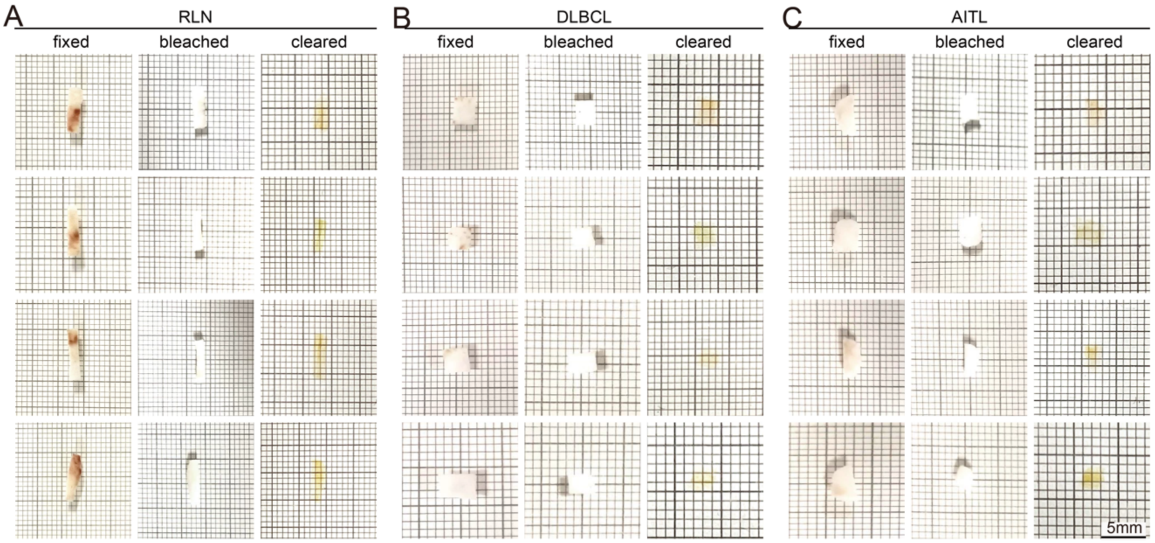

Figure S7. Photographs of RLN (A), DLBCL (B) and AITL (C) tissue blocks after fixation, bleaching, and clearing.

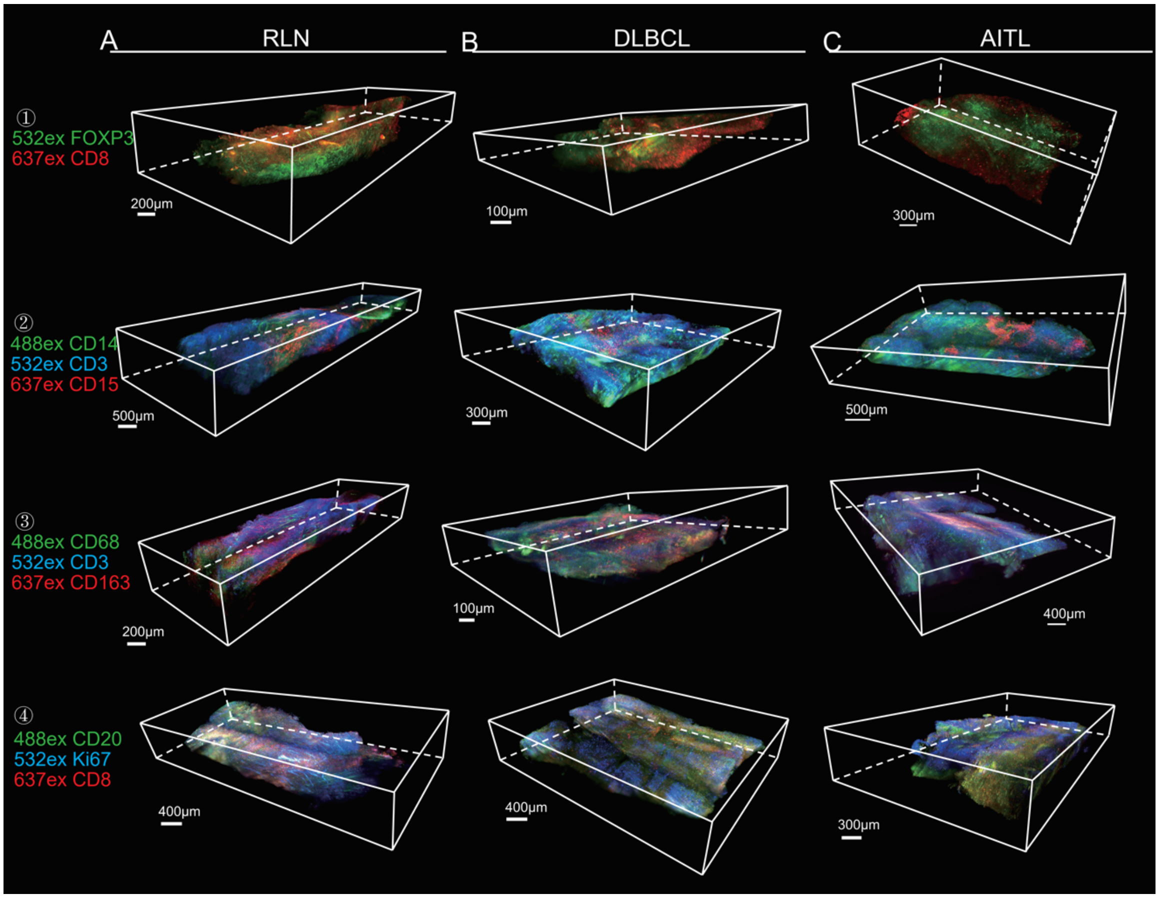

Figure S8. 3D volume rendering of the immune TMEs in cleared RLN (A), DLBCL (B) and AITL tissues (C).

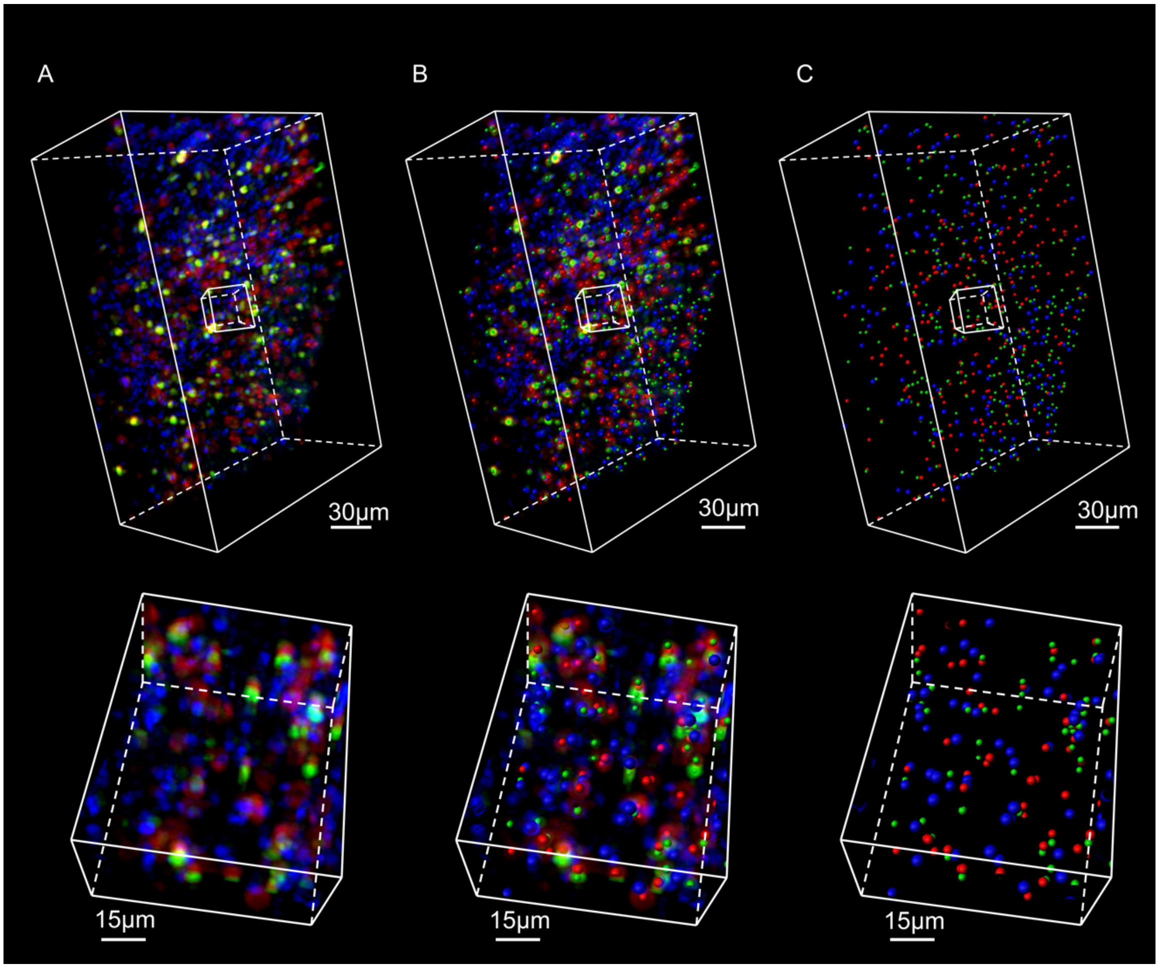

Figure S9. Cell localization and counting by Imaris software. (A) Multiplexed 3D image by LSFM. (B) Spot function in Imaris identifying the centers of the cells. (C) Counting and localization based on the segmented cells.
